# Identification of minimal human MHC-restricted CD8+ T-cell epitopes within the *Plasmodium falciparum* circumsporozoite protein (CSP)

**DOI:** 10.1186/1475-2875-12-185

**Published:** 2013-06-05

**Authors:** Martha Sedegah, Yohan Kim, Harini Ganeshan, Jun Huang, Maria Belmonte, Esteban Abot, Jo Glenna Banania, Fouzia Farooq, Shannon McGrath, Bjoern Peters, Alessandro Sette, Lorraine Soisson, Carter Diggs, Denise L Doolan, Cindy Tamminga, Eileen Villasante, Michael R Hollingdale, Thomas L Richie

**Affiliations:** 1 US Military Malaria Vaccine Program, Naval Medical Research Center, Walter Reed Army Institute of Research, Silver Spring, MD, USA; 2 La Jolla Institute for Allergy and Immunology, La Jolla, CA, USA; 3 Queensland Institute of Medical Research, Brisbane, Queensland, Australia; 4 USAID, Washington, DC, USA

**Keywords:** Malaria, Vaccine, Circumsporozoite protein, ELISpot, Flow cytometry, NetMHC, Epitope mapping, Class I restriction, Localization

## Abstract

**Background:**

*Plasmodium falciparum* circumsporozoite protein (CSP) is a leading malaria vaccine candidate antigen, known to elicit protective antibody responses in humans (RTS,S vaccine). Recently, a DNA prime / adenovirus (Ad) vector boost vaccine encoding CSP and a second *P*. *falciparum* antigen, apical membrane antigen-1, also elicited sterile protection, but in this case associated with interferon gamma ELISpot and CD8+ T cell but not antibody responses. The finding that CSP delivered by an appropriate vaccine platform likely elicits protective cell-mediated immunity provided a rationale for identifying class I-restricted epitopes within this leading vaccine candidate antigen.

**Methods:**

Limited samples of peripheral blood mononuclear cells from clinical trials of the Ad vaccine were used to identify CD8+ T cell epitopes within pools of overlapping 15mer peptides spanning portions of CSP that stimulated recall responses. Computerized algorithms (NetMHC) predicted 17 minimal class I-restricted 9-10mer epitopes within fifteen 15mers positive in ELISpot assay using PBMC from 10 HLA-matched study subjects. Four additional epitopes were subsequently predicted using NetMHC, matched to other study subjects without initial 15mer ELISpot screening. Nine of the putative epitopes were synthesized and tested by ELISpot assay, and six of these nine were further tested for CD8+ T cell responses by ELISpot CD4+ and CD8+ T cell-depletion and flow cytometry assays for evidence of CD8+ T cell dependence.

**Results:**

Each of the nine putative epitopes, all sequence-conserved, recalled responses from HLA-matched CSP-immunized research subjects. Four shorter sequences contained within these sequences were identified using NetMHC predictions and may have contributed to recall responses. Five (9-10mer) epitopes were confirmed to be targets of CD8+ T cell responses using ELISpot depletion and ICS assays. Two 9mers among these nine epitopes were each restricted by two HLA supertypes (A01/B07; A01A24/A24) and one 9mer was restricted by three HLA supertypes (A01A24/A24/B27) indicating that some CSP class I-restricted epitopes, like DR epitopes, may be HLA-promiscuous.

**Conclusions:**

This study identified nine and confirmed five novel class I epitopes restricted by six HLA supertypes, suggesting that an adenovirus-vectored CSP vaccine would be immunogenic and potentially protective in genetically diverse populations.

## Background

The circumsporozoite protein (CSP) is the main antigenic component of several candidate malaria vaccines, including the RTS,S vaccine currently undergoing Phase 3 testing in sub-Saharan Africa. RTS,S induces anti-CSP antibodies thought to mediate protection by targeting sporozoites, inhibiting motility and hepatocyte invasion [1]. This proposed mechanism is supported by the finding that both antibody and CD4+ T cell responses to CSP correlate with protection [1]. CD8+ T cell responses, however, have not been consistently demonstrated in individuals vaccinated with RTS,S [1,2]. Thus it is not clear whether RTS,S additionally targets the liver stages of *Plasmodium*, where immunity appears to be CD8+ T cell dependent [3]. CSP is carried into hepatocytes by invading sporozoites [4] and is expressed as peptides on the surface of the infected hepatocytes in the context of MHC Class I, potentially allowing recognition by CSP-specific CD8+ T cells [5]. Vaccine platforms such as adenovirus vectors promoting the induction of CD8+ T cell responses to CSP or other antigens expressed on the surface of infected hepatocytes might therefore improve protection against liver stage parasites [6].

To this aim, a replication-deficient adenovirus (Ad)-vectored vaccine encoding *Plasmodium falciparum* CSP (*Pf*CSP) (NMRC-MV-Ad-PfC, or Ad-C) was tested in humans. The vaccine, based on human adenovirus serotype 5, was tested by itself and in combination with a second Ad vector encoding apical membrane antigen-1 (AMA1) (NMRC-MV-Ad-PfA, or Ad-A). Both Ad-C and Ad-CA (the combination of Ad-C and Ad-A) vaccines elicited robust CD8+ T cell responses against both antigens [7-9], similar to other Ad-based vaccines under development for different pathogens [10]. AMA1 was added to CSP because AMA1 is likewise involved in hepatocyte invasion by the malaria parasite [11], and AMA1 peptides may similarly be expressed on the surface of infected hepatocytes. Simultaneous expression of peptides derived from both antigens could facilitate targeting by effector CD8+ T cells. To further enhance cell-mediated responses, the combination Ad-CA vaccine was primed with three doses of DNA expressing CSP and AMA1. The resulting DNA/Ad regimen induced sterile protection against controlled human malaria infection (CHMI) in 27% of immunized volunteers, with protection significantly correlated with CD8+ T cell interferon-gamma responses [12]. Antibody responses were relatively low, and did not correlate with protection, consistent with the hypothesis that CD8+ T cells may be the primary immune effector targeting liver stage parasites in humans [3].

The aim of the current study was to identify the class I CD8+ T cell epitopes in CSP recognized by the cell-mediated responses to the Ad-C and Ad-CA vaccines. Such epitopes could be used in the design of epitope-based vaccines, and responses to these epitopes could be assessed prospectively as potential correlates of protection induced by the DNA/Ad vaccine or other CSP-based vaccines. Previously, 14 class I-restricted epitopes were identified within AMA1, using a combination of predictive algorithms (NetMHC [13]) and cellular immunoassays [14]. The current study applied similar methods to map class I-restricted epitopes in CSP.

**Figure 1:**
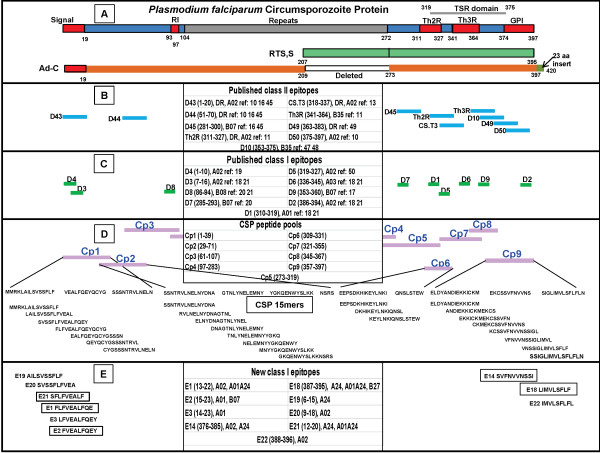
***Plasmodium falciparum *CSP: structure, vaccines and distribution of class I and class II-restricted epitopes.** The structure of *P*. *falciparum* CSP (3D7) comprises of N- and C-terminal regions flanking a central repeat region (Panel **A**). The N-terminal region contains a conserved region R1, and the C-terminal region contains variable T epitope regions Th2R and Th3R that are contained within the thrombospondin-like type I repeat (TSR), and ends in a region attached to the GPI anchor. The RTS,S vaccine contains part of the repeats and the C-terminal region; the Ad-C vaccine contains full length CSP, except for a deletion of 16 NANP repeats (64 aa) leaving 26 repeats intact and for an insertion of a 23 aa tail at the C terminus (derived from the 3’-noncoding bovine growth hormone polyadenylation sequence). Published class II (Panel **B**) and class I (Panel **C**) epitopes occur in the N- and C-terminals. The sequences for the CSP peptide pools Cp1-Cp9 and the individual 15mers within each of four dominant pools are listed (Panel **D**). Amino acids 1–39, 29–71, 309–331 and 357–397, corresponding to the four immunodominant pools, were included in the analysis. Amino acids 72–308 and 332–356 were excluded. Panel E shows the new predicted class I-restricted epitopes derived from the Cp1, Cp2, Cp6 and Cp9 15mers. Those that were confirmed in ELISpot depletion or ICS assays are boxed (Panel **E**).

*Pf*CSP (3D7 strain) contains 397 amino acids (aa), with the N-terminal region spanning aa 1–104, the central repeat region spanning aa 105–272, and the C-terminal region spanning aa 273–397 (Figure 1). The C-terminal region contains the thrombospondin-like type 1 repeat domain [15] overlapping the Th2R and Th3R T epitope regions [16]. While the RTS,S vaccine contains aa 207–395 [17], and therefore lacks the N-terminal region [18], the Ad-C vaccine is full length except for a deletion of 16 repeats (64 aa) between 209–272 (leaving 26 repeats intact), and the insertion of a 23 aa tail at the C-terminus, derived from the 3’-noncoding bovine growth hormone polyadenylation sequence [7,8].

A summary of previously published DR-restricted epitopes identified in CSP is shown in Figure 1. These DR-restricted epitopes were identified using malaria-endemic volunteers or volunteers immunized with radiation-attenuated sporozoites (RAS). Three DR-restricted epitopes, D43, D44 and D50 [19-21], were originally characterized using class II binding assays to identify peptides that recalled proliferative responses from individuals living in a malaria-endemic area; D10, D45, D49, Th2R and Th3R [22] were characterized using overlapping peptides to recall proliferative responses, likewise from individuals living in malaria-endemic areas; and CS.T3 was identified using proliferation assays testing samples from volunteers immunized with RAS [23].

Previously published class I-restricted CSP epitopes are also shown in Figure 1, and have been identified in both the N- and C-terminal regions. D1, D2, D3, and D6 were identified using class I binding assays to identify peptides that induced cytotoxic recall activities from volunteers immunized with RAS and from malaria-endemic volunteers [24,25]; D4 was identified using peptides to recall proliferation responses from malaria-endemic volunteers [26]; and D5, D7, D8 and D9 were characterized using short peptides designed to match the HLA of malaria-endemic volunteers [27,28]. D5 and D9 have been tested in mice for induction of cytotoxic T cell responses [29]. More recently, D4, D5 and D6 were shown to recall interferon-gamma responses in ELISpot assays using PBMCs from individuals living in a malaria-endemic region of Ghana [30].

HLA alleles have been grouped into nine supertypes that are clusters of alleles with similar peptide-binding motifs [31], and the majority of HLA alleles fit these supertypes [31-33]. Based on algorithms that predict binding to MHC molecules, measured as 50% inhibitory concentration (IC_50_) values expressed as nanomolar (nM) [34], a meta-analysis using an affinity cut-off of 500 nM predicted that 52% of a panel *P*. *falciparum* peptides bound to HLA A*02:01 [35], and led to the development of publically available algorithms that are specific for class I and class II types [35]. The outcomes of these and similar studies led to the establishment of the Immune Epitope Database and Analysis Resource (IEDB) that contains open access data and analytical tools for malaria and a wide range of other organisms [36]. Class II-restricted epitopes are well known to be promiscuous, binding to multiple HLA alleles [37], including DR-restricted epitopes in CSP [21]. Similar promiscuity in class I-restricted epitopes has been described for malaria antigens including CSP [24] and has been extended to include epitopes from other organisms [38,39]. Recently, analysis of the IEDB data base suggests that >50% of HLA class I-restricted ligands bind to two or more HLA molecules often spanning different supertypes [40].

To conduct the mapping studies of CSP epitopes, peripheral blood mononuclear cells (PBMC) were selected from limited supplies of frozen specimens previously collected from volunteers immunized with Ad-C or Ad-CA in three different trials [7-9]. Nine CSP peptide pools containing three to 12 overlapping 15mer peptides had been used during the original analysis of these clinical trials to characterize responses in ELISpot assays and flow cytometry, with four pools giving the highest responses [7]. Since the numbers of frozen PBMC from these three trials were limited, only these four CSP peptide pools were used for epitope mapping, and for the same reason (limited PBMC supplies), previously described class I epitopes were not tested.

## Methods

### Vaccines and trial design

**Figure 2:**
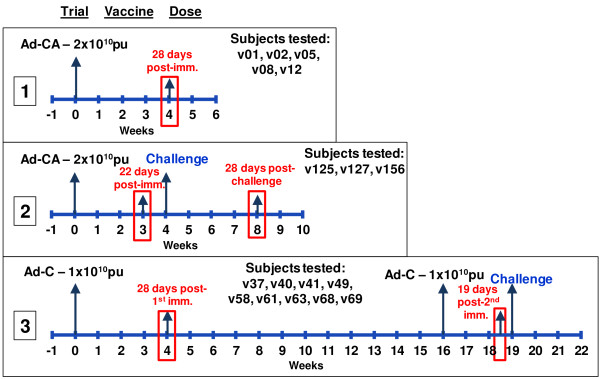
**Vaccine trials with adenovirus vectored CSP used to map CSP epitopes.** Three trials using adenovirus-vectored CSP (Ad-C) were performed: trial 1 used Ad-C combined with adenovirus-vectored AMA (Ad-A) given as one immunization (Ad-CA) to six subjects without CHMI; trial 2 used the same dose of Ad-CA given to 18 subjects followed by CHMI four weeks later; trial 3 used Ad-C administered twice 16 weeks apart (to 15 and 14 subjects respectively) followed by CHMI four weeks later. Collection points for PBMC used in this study are shown in boxes. The 17 subjects that were used in this study are identified by trial. Challenge=CHMI.

The vaccine used in this study was either the CSP-encoding Ad vector alone (Ad-C) or the same in combination with AMA1-encoding Ad vector (Ad-CA), in three different clinical trials (Figure 2). In the first trial, six volunteers were immunized with 2 × 10^10^ particle units (pu) of both adenovectors (Ad-CA) as a single intramuscular dose, but there was no controlled human malaria infection (CHMI) to determine efficacy in this small safety study [7]. In the second trial, 18 volunteers were similarly immunized once with 2 × 10^10^ pu of Ad-CA, and in this trial they underwent CHMI by bite of *P*. *falciparum*-infected mosquitoes [9]. In the third trial, volunteers were immunized twice with 1 × 10^10^ pu of Ad-C alone at week 0 (15 volunteers) and week 16 (14 volunteers). These research subjects also underwent CHMI by bite of *P*. *falciparum*-infected mosquitoes [8]. While none of the volunteers in the two challenge studies was sterilely protected against malaria (contrasting with the trial where DNA was first used to prime the response), the Ad-alone vaccine regimens were strongly immunogenic for CD8+ and CD4+ T cell responses.

### Volunteers and HLA typing

HLA molecular typing for HLA-A and HLA-B loci was performed by the Department of Defense Bone Marrow Donor Program using specific oligonucleotide probes to amplify HLA Class I genes. Typing included a list of allelic codes from which it was possible to assign each volunteer to an HLA-A or HLA-B allele group using code lists as previously described [14]. Each HLA-A or HLA-B allele group was then assigned to HLA A or HLA B supertypes according to published nomenclatures. All 17 volunteers from the three trials used in this study, and their HLA A and B allele groups, are shown in Table 1.

**Table 1:** Volunteer HLA A and B allele groups and supertypes

**Vaccine**	**Vol.**	**Strategy**			**Trial**	**HLA-A1 allele group**	**HLA-A2 allele group**	**HLA-B1 allele group**	**HLA-B2 allele group**	**HLA-A1 supertype**	**HLA-A2 supertype**	**HLA-B1 supertype**	**HLA-B2 supertype**
		**1A**	**1B**	**2**									
Ad-CA	v1	**x**			**1**	A*02:01	A*26:01	B*18:01	B*44:02	A02	A01	B44	B44
	v2	**x**			**1**	A*01:01	A*02:01	B*08:01	B*44:02	A01	A02	B08	B44
	v5	**x**			**1**	A*01:01	A*68:02	B*08:01	B*14:02	A01	A02	B08	B27
	v8	**x**			**1**	A*68:01	A*68:02	B*14:02	B*48:01	A03	A02	B27	B27
	v12	**x**			**1**	A*30:02	A*68:01	B*18:01	B*58:02	A01	A03	B44	B58
Ad-CA	v125			**x**	**2**	A*02:01	A*11:01	B*35:01	B*52:01	A02	A03	B07	B62
	v127		**x**		**2**	A*01:01	A*24:02	B*08:01	B*44:05	A01	A24	B08	B44
	v156		**x**	**x**	**2**	A*03:01	A*29:02	B*15:03	B*58:02	A03	A01 A24	B27	B58
Ad-C	v37		**x**	**x**	**3**	A*23:01	A*68:02	B*15:03	B*53:01	A24	A02	B27	B07
	v40		**x**	**x**	**3**	A*23:01	A*29:02	B*52:01	B*53:01	A24	A01	B62	B07
	v41	**x**			**3**	A*02:01	A*31:01	B*07:02	B*35:01	A02	A03	B07	B07
	v49	**x**			**3**	A*33:01	A*74:01	B*15:03	B*15:03	A03	A03	B27	B27
	v58	**x**	**x**		**3**	A*02:01	A*24:02	B*08:01	B*38:02	A02	A24	B08	B27^1^
	v61	**x**			**3**	A*02:01	A*02:01	B*38:01	B*44:02	A02	A02	B27	B44
	v63			**x**	**3**	A*11:01	A*24:03	B*40:01	B*51:04	A03	A24	B44	B07
	v68			**x**	**3**	A*24:02	A*30:01	B*13:02	B*14:02	A24	A01 A03	B62^1^	B27
	v69	**x**	**x**		**3**	A*30:02	A*34:02	B*14:02	B*35:01	A01	A03	B27	B07

The volunteers from whom PBMC were available and were tested are shown. Trial 1 (no CHMI): five of six volunteers immunized with Ad-CA were used (reference 7). Trial 2 (with CHMI): three of 17 volunteers immunized with Ad-CA and challenged by bite of *P. falciparum*-infected mosquitoes were used (Tamminga, in press). Trial 3 (with CHMI): nine of 11 volunteers immunized with Ad-C and challenged by bite of *P. falciparum*-infected mosquitoes were used (reference [8]). The table also identifies the ten volunteers with best available PBMC that were included in the broad screen of all 15-mer peptides from the four dominant pools (column 1A), the six volunteers used to confirm recognition of minimal epitopes predicted within positive 15-mers (column 1B) (1A+1B=strategy 1), and the six volunteers used to confirm recognition of minimal epitopes predicted within 15-mers without the initial screen (strategy 2).

^1^As classified in reference [42].

### Peripheral blood mononuclear cells (PBMC)

The PBMC used in this study for epitope mapping were collected 19–28 days following Ad administration, a period corresponding to the peak response. In a few cases where samples were insufficient, collections from 28 days post challenge (56 days post Ad administration) were used instead, as responses to this vaccine in a prior study persisted for at least 12 months in most research subjects [7]. In summary, the PBMC samples were obtained from the following time points: trial 1 (Ad-CA, no CHMI), 28 days following immunization; trial 2 (Ad-CA, with CHMI), 22–23 days after immunization and 28 days after challenge; trial 3 (Ad-C administered twice followed by CHMI), 28 days and 19 days after the first and second immunizations, respectively. These time points are indicated in Figure 2 and, where appropriate, in the Tables (see below). Previous studies have confirmed that recall T cell responses measured by ELISpot assay are able to be detected using cryopreserved PBMCs although such responses are generally of lower magnitude than fresh cells [14].

### Peptides and peptide pools

**Figure 3:**
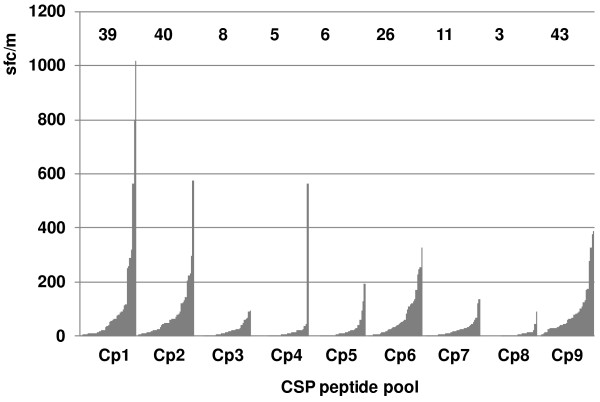
**ELISpot activity of CSP peptide pools with all volunteers in adenovirus-vectored CSP trials.** The ELISpot activities of all subjects from three adenovirus-vectored CSP trials (see Figure 2) recalled by each CSP peptide pool (Cp1-Cp9) were arranged in ascending order of activity. The numbers above each peptide pool are the geometric means of activities to that pool. Cp1, Cp2, Cp6 and Cp9 were selected for this study as the immunodominant pools.

Sixty-five 15mer peptides overlapping by 11 amino acids and spanning the full length of CSP (3D7 strain) were synthesized commercially (Mimotopes, VIC, Australia, >80% purity) and grouped into nine peptide pools containing three to 12 peptides in each (Figure 1). Four of these pools (Cp1, Cp2, Cp6, and Cp9) containing 26 peptides (Table 2) elicited the highest ELISpot responses among the volunteers who received the Ad-C or Ad-CA vaccines [7] (Figure 3) and were selected for this study. Minimal (9-10mer) epitopes were synthesized by Alpha Diagnostics Intl Inc, San Antonio, TX, USA (>91% purity).

**Table 2:** CSP peptides used in ELISPOT and ICS assays

**Pool**	**Amino acids**	**15mer**	**Amino acids**	**Sequence**
Cp1	1 - 39	C1	1-15	MMRKLAILSVSSFLF
		C2	5-19	LAILSVSSFLFVEAL
		C3	9-23	SVSSFLFVEALFQEY
		C4	13-27	FLFVEALFQEYQCYG
		C5	17-31	EALFQEYQCYGSSSN
		C6	21-35	QEYQCYGSSSNTRVL
		C7	25-39	CYGSSSNTRVLNELN
Cp2	29-71	C8	29-43	SSNTRVLNELNYDNA
		C9	33-47	RVLNELNYDNAGTNL
		C10	37-51	ELNYDNAGTNLYNEL
		C11	41-55	DNAGTNLYNELEMNY
		C12	45-59	TNLYNELEMNYYGKQ
		C13	49-63	NELEMNYYGKQENWY
		C14	53-67	MNYYGKQENWYSLKK
		C15	57-71	GKQENWYSLKKNSRS
Cp6	309-331	C46	309-323	EEPSDKHIKEYLNKI
		C47	313-327	DKHIKEYLNKIQNSL
		C48	317-331	KEYLNKIQNSLSTEW
Cp9	357-397	C58	357-371	ELDYANDIEKKICKM
		C59	361-375	ANDIEKKICKMEKCS
		C60	365-379	EKKICKMEKCSSVFN
		C61	369-383	CKMEKCSSVFNVVNS
		C62	373-387	KCSSVFNVVNSSIGL
		C63	377-391	VFNVVNSSIGLIMVL
		C64	381-395	VNSSIGLIMVLSFLF
		C65	383-397	SSIGLIMVLSFLFLN

Four CSP peptide pools, Cp1, Cp2, Cp6, and Cp9 were selected as they elicited the highest responses overall in ELISpot assays with Ad-CA-immunized volunteers (see reference [7] and Figure 3).

### Strategies to identify class I-restricted CSP epitopes

Two strategies were used to identify class I epitopes within these four immunodominant pools.

**Strategy 1:** Each of the 26 15mers contained within the four pools was tested individually by ELISpot assay using PBMC from 10 selected volunteers with sufficient PBMC available. 15mers positive by this screen were analysed by NetMHC to identify putative class I-restricted epitopes, some of which were then synthesized (see below). Depending upon PBMC availability, the putative epitopes were tested in HLA-matched volunteers by ELISpot assays for ability to recall interferon-gamma responses.

**Strategy 2:** Because there were not sufficient frozen PBMC available to extend the analysis using the comprehensive ELISpot screening with 26 15mers to additional volunteers, NetMHC predictions were used directly to identify class I-restricted epitopes within 15mers that could be matched to six additional volunteers with robust responses against the parent pool. Some of these minimal epitopes were also synthesized and tested in ELISpot assays. As a control, some predicted epitopes were tested with non-HLA-matched volunteers for whom PBMC were available.

### *Ex vivo* IFN-γ enzyme-linked immunospot (ELISpot) assays

IFN-γ ELISpot assays were conducted as previously described [14,41]. Cryopreserved PBMC were suspended in 100 μL complete medium and stimulated with CSP peptides in 100 μL of complete medium at a final concentration of 10 μg/mL of each peptide tested [41]. Cultures were incubated for 36 hours at 37°C, 5% CO2. Each PBMC sample was assayed in duplicate, triplicate, or quadruplicate and the number of IFN-γ-secreting spot forming cells (sfc) was counted using an automated ELISpot reader (AID, GmbH, Germany). The positive control was commercially obtained Class I Peptide Pool *Plus* (CEF; Anaspec, USA) that stimulates IFN-γ from CD8+ T cells [42]. Negative control was media with all supplements except antigen-specific stimulants. In duplicate assays, both values were used in the analysis. For triplicate or quadruplicate assays, outliers were rejected if any single value contributed more than 50% of the standard deviation of the replicates and if its value was three-fold greater or three-fold less than the average of the remaining two (or three) values. The mean number of sfc obtained in negative control wells was subtracted from the value of each test well from the same sample. Negative counts generated by this background subtraction were converted to zero. The mean number of spots of the test sample was then calculated and expressed as sfc/million (sfc/m). A positive response was defined as a significant difference (p = <0.05) between the average of sfc in test wells and negative control wells (Student’s two tailed *t*-test), plus at least a doubling of sfc in test wells relative to negative control wells, plus a difference of at least 10 sfc between test and negative control wells [7].

### Characterization of ELISpot IFN-γ-producing cells by T-cell subset depletions

PBMC were depleted of T-cell subsets using anti-human CD4+ or anti-CD8+ coated Dynabeads M-450 (Dynal, Great Neck, NY, USA) following the manufacturer’s instructions as previously described [7]. Mock depletion was done with Dynabeads coated with sheep anti-mouse IgG. Flow cytometry confirmed that T-cell subset depletions were >99% in all experiments. Data are presented as sfc/m and per cent decrease or increase in activity after depletion.

### Intracellular cytokine staining (ICS)

ICS was performed as published previously [14]. Cryopreserved PBMC were thawed, washed, and resuspended at 1×10^6^ cells per mL in complete medium. Peptides were used at 10 μg/mL and costimulatory antibodies anti-CD28 and anti-CD4 + 9d (BD Bioscience, San Jose, CA, USA) were used at 1 μg/mL. Stimulants were added to cells and incubated at 37°C with 5% CO_2_ for two hours. Cells were stained with anti-CD3, anti-CD4+, anti-CD8+, anti-IFN-γ, anti-TNFα, and anti-IL2 and the entire available sample was acquired on a BD LSRII using FACSDiVa (BD Bioscience) software. Data were analysed using FlowJo software (Treestar, Inc). The gating strategy involved progressively measuring total cells; viable cells; lymphocytes; T cells; CD4+ or CD8+ populations; and finally a specific cell type expressing a specific cytokine. Results were transferred to Prism (GraphPad) for graphing and data were corrected for media responses. A positive response was greater than the medium controls + three standard deviations (0.03%).

### NetMHC-based epitope predictions

NetMHC [13] was used to predict the MHC class 1 binding affinities, expressed as the half maximum inhibitory concentration (IC_50_) of minimal 8-10mer epitopes within the 15mers that matched the HLA alleles expressed by the volunteers used in each assay. Peptides with predicted IC_50_ binding affinities less than 500 nM were considered strong binders, those 500–5,000 nM were considered weak binders, and those >5,000 nM was considered non-binders [43].

## Results

### Volunteers

A total of 17 immunized volunteers were used in all experiments (Table 1): five of six volunteers immunized with Ad-CA in trial 1 [7]; three of 18 volunteers immunized with Ad-CA in trial 2 ([9] and nine of 11 volunteers immunized with Ad-C in trial 3 (Table 1). These volunteers expressed a total of 17 different HLA A allele groups representing all HLA A supertypes [33,44] and 17 HLA B allele groups representing all HLA B supertypes (Table 1).

### ELISpot activity of CSP 15mers with volunteers immunized with Ad-C or Ad-CA

The hypothesis was that NetMHC would predict minimal binding epitopes (affinity <5,000 nM) in individual 15mers positive in ELISpot assay that were restricted by HLA allele groups that matched the HLA expressed by each volunteer. Each of the 26 15mers within each of the four dominant pools (Table 2) was used as a stimulant in individual ELISpot assays conducted using PBMC from five volunteers immunized with Ad-CA (v01, v02, v05, v08 and v12) (no CHMI trial) and five volunteers immunized with Ad-C (v41, v49, v58, v61 and v69). No volunteers from the Ad-CA with CHMI trial had sufficient PBMC to be included. All results are shown in Additional file 1 and the positive results are shown in Table 3.

**Table 3:** ELISpot IFN-γ activity of CSP peptide pools and individual 15-mer peptides within these pools

**Pool**	**Vol.**	**Vaccine**	**15mer peptide**	**AA no.**	**Sequence**	**Pool sfc/m**	**15mer sfc/m**	**Percent of pool**
**Cp1**	58	Ad-C	C3	9-23	SVSSFLFVEALFQEY	116	29	25.0
**Cp1**	05	Ad-CA	C3		SVSSFLFVEALFQEY	142	65	45.8
**Cp1**	69	Ad-C	C3		SVSSFLFVEALFQEY	411	385	93.7
**Cp1**	12	Ad-CA	C4	13-27	FLFVEALFQEYQCYG	64	87	135
**Cp1**	05	Ad-CA	C4		FLFVEALFQEYQCYG	142	44	31.0
**Cp1**	01	Ad-CA	C4		FLFVEALFQEYQCYG	77	48	62.3
**Cp1**	69	Ad-C	C4		FLFVEALFQEYQCYG	411	368	89.5
**Cp1**	58	Ad-C	C5	17-31	EALFQEYQCYGSSSN	116	105	90.5
**Cp1**	58	Ad-C	C6	21-35	QEYQCYGSSSNTRVL	116	83	71.6
**Cp2**	49	Ad-C	C8	29-43	SSNTRVLNELNYDNA	64	41	64.1
**Cp2**	41	Ad-C	C10	37-51	ELNYDNAGTNLYNEL	83	35	42.2
**Cp2**	49	Ad-C	C12	45-59	TNLYNELEMNYYGKQ	64	28	43.8
**Cp2**	12	Ad-CA	C12		TNLYNELEMNYYGKQ	331	411	124
**Cp2**	01	Ad-CA	C12		TNLYNELEMNYYGKQ	119	119	100
**Cp2**	01	Ad-CA	C13	49-63	NELEMNYYGKQENWY	119	116	97.5
**Cp2**	12	Ad-CA	C13		NELEMNYYGKQENWY	331	334	100
**Cp6**	61	Ad-C	C47	313-327	DKHIKEYLNKIQNSL	53	48	90.6
**Cp6**	41	Ad-C	C48	317-331	KEYLNKIQNSLSTEW	95	103	108
**Cp6**	05	Ad-CA	C48		KEYLNKIQNSLSTEW	130	106	81.5
**Cp6**	58	Ad-C	C48		KEYLNKIQNSLSTEW	24	45	187
**Cp9**	61	Ad-C	C60	365-379	EKKICKMEKCSSVFN	128	39	30.5
**Cp9**	01	Ad-CA	C62	373-387	KCSSVFNVVNSSIGL	39	116	297
**Cp9**	08	Ad-CA	C63	377-391	VFNVVNSSIGLIMVL	142	109	76.8
**Cp9**	08	Ad-CA	C64	381-395	VNSSIGLIMVLSFLF	142	91	64.1
**Cp9**	01	Ad-CA	C65	383-397	SSIGLIMVLSFLFLN	39	46	117
**Cp9**	02	Ad-CA	C65		SSIGLIMVLSFLFLN	16	44	275
**Cp9**	08	Ad-CA	C65		SSIGLIMVLSFLFLN	142	62	43.7

All individual 15mer peptides within the CSP peptide pools Cp1, Cp2, Cp6 and Cp9 were tested in ELISpot assay using frozen PBMC collected 28 days after Ad-CA or 19-23 days after Ad-C immunization. 15 individual 15mer peptides of the 26 15mer peptides elicited positive recall responses from at least one volunteer immunized with Ad-CA and Ad-C, and each of the ten volunteers responded positively to at least one 15mer.

Fifteen of the 26 tested 15mers were positive by ELISpot assay (Table 3). Although responses varied, it appeared that the inclusion of the AMA1 in the Ad-CA vaccine did not interfere with CSP epitope recognition by PBMC. As previously seen with NetMHC predictions [33], about 25% of the 15mers containing putative HLA-matched epitopes with predicted IC50’s <5,000 nM gave positive responses (see Additional file 1). No 15mer containing a predicted epitope was positive with all HLA-matched volunteers, and no volunteer was always positive with all 15mers containing predicted HLA-matched epitopes. When the activity of each positive 15mer was calculated as a per cent of the parent pool activity, these varied from 25 – 297%, with percentages < 100% suggesting that the parent pool may contain other 15mers able to recall responses, and percentages > 100% suggesting that the various 15mers in the parent pool may not have been fully processed and presented.

### NetMHC prediction of class I-restricted epitopes within CSP 15mer peptides

NetMHC predicted 17 HLA-matched minimal epitopes from the fifteen positive 15mers active in the ELISpot assay (Table 4), of which 11 were predicted to be strong binders (IC_50_ < 500 nM) and five were predicted to be weak binders (IC_50_ 500-5,000 nM). NetMHC identified one epitope (E5) where the IC_50_ (>5,000 nM) was too low to confirm class I binding (>5,000 nM). The predicted putative epitopes were numbered E1–E17 in sequence from the N-terminal end of CSP. The predicted epitopes included seven 9mers (E2, E6, E7, E8, E10, E12, E13) and ten 10mers (E1, E3, E4, E5, E9, E11, E14, E15, E16, E17). Eleven predicted epitopes were from the N-terminal region (E1 to E11) and six epitopes were from the C-terminal region (E12 to E17). Potential putative epitopes located in regions of the CSP not covered by the four dominant pools (see Figure 1) were not considered in this study.

**Table 4:** **Predicted CD8**+ **T cell**-**restricted epitopes specific for each volunteer within CSP 15mer peptides**

**Pool**	**15mer**	**Vol.**	**Predicted epitope**	**AA no.**	**IC**_**50**_**nM**	**HLA allele group**	**HLA supertype**	**Epitope no.**
Cp1	C3	58	SVSS**FLFVEALFQE**Y	13-22	258	A*02:01	A02	E1
		05	SVSSFL**FVEALFQEY**	15-23	50	A*01:01	A01	E2
		69	SVSSFL**FVEALFQEY**	15-23	68	B*35:01	B07	E2
	C4	12	F**LFVEALFQEY**QCYG	14-23	226	A*30:02	A01	E3
		01	**FLFVEALFQE**YQCYG	13-22	258	A*02:01	A02	E1
		69	FL**FVEALFQEY**QCYG	15-23	68	B*35:01	B07	E2
	C5	58	E**ALFQEYQCYG**SSSN	18-27	2174	A*02:01	A02	E4
	C6	58	QE**YQCYGSSSNT**RVL	23-32	11714	A*02:01	A02	E5
Cp2	C8	49	SSN**TRVLNELNY**DNA	32-40	4075	B*15:03	B27	E6
	C10	41	ELN**YDNAGTNLY**NEL	40-48	321	B*35:01	B07	E7
	C12	49	**TNLYNELEMNYY**GKQ	45-53	1087	B*15:03	B27	E8
		12	TN**LYNELEMNYY**GKQ	47-56	25	A*30:02	A01	E9
		01	TNLY**NELEMNYYG**KQ	49-57	468	B*44:02	B44	E10
	C13	12	NELEM**NYYGKQENWY**	54-63	132	A*30:02	A01	E11
Cp6	C47	61	DKHIKE**YLNKIQNSL**	319-327	27	A*02:01	A02	E12
	C48	41	KE**YLNKIQNSL**STEW	319-327	27	A*02:01	A02	E12
		05	KE**YLNKIQNSL**STEW	319-327	83	B*08:01	B08	E12
		58	KE**YLNKIQNSL**STEW	319-327	27	A*02:01	A02	E12
Cp9	C60	61	EKKICK**MEKCSSVFN**	371-379	2353	B*44:02	B44	E13
	C62	01	KCS**SVFNVVNSSI**GL	376-385	470	A*02:01	A02	E14
	C63	08	VF**NVVNSSIGLI**MVL	379-388	70	A*68:02	A02	E15
	C64	08	V**NSSIGLIMVL**SFLF	382-391	294	A*68:02	A02	E16
	C65	01	SSIG**LIMVLSFLFL**N	387-396	53	A*02:01	A02	E17*
		02	SSIG**LIMVLSFLFL**N	387-396	53	A*02:01	A02	E17*
		08	SSIG**LIMVLSFLFL**N	387-396	816	A*02:01	A02	E17*

The 15mer peptides that were recognized by the volunteers in the initial screen (Table 2) were analysed by NetMHC to predict affinity HLA binding by minimal CD8+ T cell epitopes within each 15-mer (underlined and bold). Those minimal epitopes with the strongest binding affinities for the HLA alleles of each volunteer were selected. Each minimal epitope was specific for a known HLA allele within each supertype. Two listings from Table 4 were not included here because the same minimal peptide was predicted for the same volunteer for two overlapping 15mers (v01, C12 and C13; v05, C3 and C4). Nine of these epitopes were synthesized and further screened by ELISpot with HLA-matched volunteers (see Table 5).

*E17, a 10-mer, was predicted to contain a 9mer sequence LIMVLSFLF labelled E18 (see Table 5).

Vol. = volunteer; AA = amino acid; No. = number.

### HLA promiscuity of predicted epitopes within 15mers

Two putative epitopes were predicted to be restricted by different HLA supertypes (Table 4): E2 by both A*01:01 allele group (A01 supertype) and B*35:01 allele group (B07 supertype), and E12 by A*02:01 allele group (A02 supertype) and B*08:01 allele group (B08 supertype), supporting recent meta-analyses that >50% of IEDB-listed epitopes bind two or more HLA molecules [40]. The remaining putative epitopes were predicted to be restricted by single HLA allele groups: E1, E4, E5, E14 and E17 by A*02:01 (A02 supertype); E3, E9 and E11 by A*30:02 (A01 supertype); E15 and E16 by A*68:02 (A02 supertype); E10 and E13 by B*44:02 (B44 supertype); E6 and E8 by B*15:03 (B27 supertype); and E7 by B*35:01 (B07 supertype). Epitopes predicted to bind to supertypes A03 and B58, which were expressed by some volunteers (Table 1), were not identified by NetMHC in the 15mers positive in ELISpot from these four selected peptide pools, although this does not mean definitively that none occur.

### ELISpot activity of synthesized minimal epitope peptides with volunteers immunized with Ad-CA or Ad-C

Six volunteers were selected on the basis of HLA-matching and availability of PBMC to test in ELISpot assays five of the predicted putative epitopes identified through the comprehensive 15mer screen and NetMHC, E1, E2, E3, E14 and E18. These were v58, v69, 37 and v40 from the Ad-C trial and v127 and v156 from the Ad-CA CHMI trial. The pairing of volunteers and epitopes is shown in the top half of Table 5.

**Table 5:** **ELISpot IFN-γ activity of CSP peptide pools and predicted 8-10mer epitopes within these pools (strategy 1 and strategy 2**)

**Pool**	**Vol.**	**Epitope no.**	**Epitope sequence**	**AA no.**	**HLA allele group**	**HLA supertype**	**IC**_**50**_**nM**	**Pool sfc/m**	**Epitope sfc/m**	**Percent of pool**
**Cp1**	58^1^	**E1**	FLFVEALFQE	13-22	A*02:01	A02	258	ND	49	
**Cp1**	156^3^	**E1**	FLFVEALFQE	13-21	A*29:02	A01A24	422	126	105	83.3
		**E1**	FLFVEALFQE	13-20	A*29:02	A01A24	109			
**Cp1**	69^2^	**E2**	FVEALFQEY	15-23	B*35:01	B07	68	399	268	67.2
**Cp1**	127^3^	**E2**	FVEALFQEY	15-23	A*01:01	A01	63	69	89	129
**Cp1**	69^2^	**E3**	LFVEALFQEY	14-23	A*30:02	A01	63	ND	259	
**Cp1**	69^2^	**E3 (E2)**	LFVEALFQEY	15-23	B*35:01	B07	68	399	259	64.9
**Cp1**	127^3^	**E3 (E2)**	LFVEALFQEY	15-23	A*01:01	A01	63	69	99	144
**Cp9**	37^1^	**E14**	SVFNVVNSSI	376-385	A*68:02	A02	18	79	28	35.4
**Cp9**	58^1^	**E14**	SVFNVVNSSI	376-385	A*02:01	A02	470	13	80	615
**Cp9**	40^1^	**E14**	SVFNVVNSSI	377-385	A*23:01	A24	1801	43^4^	104, 72	242, 167
**Cp9**	156^3^	**E18**	LIMVLSFLF	387-395	A*29:02	A01A24	190	64	156	244
**Cp9**	37^1,2^	**E18**	LIMVLSFLF	387-395	B*15:03	B27	111	79^4^	55, 38	69.6, 48.1
**Cp9**	40^1^	**E18**	LIMVLSFLF	387-395	A*23:01	A24	282	43^4^	290, 166	674, 386

**Cp1**	63^1^	**E19**	AILSVSSFLF	6-15	A*24:03	A24	1088	ND	41	
**Cp1**	125^3^	**E20**	SVSSFLFVEA	9-18	A*02:01	A02	25	27	33	122
**Cp1**	68^1^	**E21**	SFLFVEALF	12-20	A*24:02	A24	104	ND	34	
**Cp1**	37^1,2^	**E21**	SFLFVEALF	12-20	A*23:01	A24	104	36^4^	53, 53	147, 147
**Cp1**	40^1^	**E21**	SFLFVEALF	12-20	A*23:01	A24	104	163^4^	313, 197	192, 121
**Cp1**	156^3^	**E21**	SFLFVEALF	12-20	A*29:02	A01A24	245	120	240	200
**Cp9**	125^3^	**E22**	IMVLSFLFL	388-396	A*02:01	A02	59	58	29	50.0

This table includes all 19 epitope-specific responses that were positive among the 35 that were tested. The presence of two results in the epitope response column (second from right) indicates that two separate experiments were performed. E18 is a 9mer sequence predicted by NetMHC within E17 (Table 4) and was synthesized rather than E17 and tested with three volunteers immunized with Ad-CA or Ad-C. Three of the 9mer sequences that are underlined indicate additional class I-restricted sequences predicted by NetMHC contained within E1 and E14 epitopes respectively. E1 was positive with both the volunteer predicted to recognize the full sequence and the volunteer predicted to recognize two nested epitopes. E14 was positive for both the two volunteers predicted to recognize the full sequence and the one volunteer predicted to recognize the nested epitope. A single assay was performed for v69 and E3; however, E3 contains the E2 sequence that is underlined and has a different predicted restriction than E3, so both are listed. E14 contains a predicted A*23:01-restricted epitope that is underlined.

^1^PBMC from 28 days after first Ad-C immunization; ^2^PBMC from 19 days after second Ad-C immunization; ^3^PBMC from 28 days after Ad-CA immunization; ^4^The response to the peptide pool was not done in the second assay.

ND, Not Done.

A second round of assays was also conducted (strategy 2) to analyse four additional predicted putative epitopes selected from positive parent 15mers, numbered E19 to E22. E19 and E20 were predicted to bind to A*24:03 and A*02:01, respectively, while E21 was predicted to bind promiscuously to A*24:02, A*23:01 and A*29:02. Like E19 and E20, E22 was predicted to bind to A*02:01. PBMC were available from six HLA-matched volunteers to test these four additional peptides, including v156 (AdCA, with CHMI), v37 (AdC) and v40 (AdC), all used to test the first set of epitopes (see above), and three new volunteers, v125 (AdCA, with CHMI), v63 (AdC) and v68 (AdC). The pairing of volunteers and these four additional putative epitopes for the conduct of ELISpot assays is shown in the bottom half of Table 5.

The positive control for each assay was the parent CSP peptide pool that contained the 15mer peptide from which these epitopes were derived (noting that the magnitude of response recalled by some parent pools was lower than that recalled by predicted epitope it contained). We also conducted six assays where the volunteer and epitope matched at the supertype but not the allele group level, and also seven assays constituting HLA mismatches.

Altogether thirty-five assays were conducted using the nine synthesized epitopes, with the positive assay results shown in Table 5, ranging from 0 to 331 sfc/10^6^ PBMC (for all results, see Additional file 2). When the activity of each positive result was calculated as a per cent of the parent pool activity, these varied from 35.4 – 674%, suggesting that the parent pool may contain other epitopes that were not tested here (<100%), or that the various epitopes in the parent pool may not have been fully processed and presented (>100%). Interestingly, nearly all assays (18/20) matching synthesized minimal epitopes to research subjects by allele group were positive. The two exceptions were E19 that matched v40 and E22 that matched v37 and were negative. All six of the assays where the epitope matched at the supertype but not the allele group level, and also all seven HLA mismatches, were negative (Additional file 2).

The findings for each of the nine epitopes were as follows:

**E1(FLFVEALFQE):** This sequence was supported as an A*02:01-restricted (A02 supertype) epitope as the synthesized peptide was active with v58 (Table 5). E1 was also positive with v156 who does not express A*02:01; however, E1 contains the 9mer **FLFVEALFQ** and the 8mer **FLFVEALF** that NetMHC predicted each bound to A*29:02 that is expressed by v156 (Table 5). Therefore, E1 is A*02:01-restricted and also contains two predicted sequences restricted by A*29:02 (both of which are listed in the table).

**E2(FVEALFQEY):** This sequence was supported as a B*35:01-restricted epitope (B07 supertype) as the synthesized peptide was active with v69 (Table 5). E2 was also predicted by NetMHC to be an A*01:01-restricted epitope (A01 supertype) using v05; however, PBMC were not available from v05 to test the synthesized peptide. E2, however, could be tested with v127, also A*01:01-restricted, and was active. Therefore E2 appears restricted by two allele groups, B*35:01 and A*01:01 that belong to different supertypes, B07 and A01, respectively.

**E3(LFVEALFQEY):** This sequence was initially identified as A*30:02-restricted, as the parent C4 15mer was active with v12 (Table 4). Since there were not sufficient PBMC from v12, E3 was tested and was active with v69, concordant with NetMHC-predicted binding to A*30:02 (A01 supertype). E3 contains the 9mer E2 sequence **(FVEALFQEY)** that NetMHC predicted binds to B*35:01 that is also expressed by v69 (see E2 above). Both potential HLA associations (A*30:02, B*35:01) for v69’s positive result (259 sfc/m) are listed in Table 5. E3 was also positive with v127, concordant with the NetMHC prediction that the E2 sequence within E3 bound to A*01:01 (see E2 above). Therefore, activities of E3 with v127 and v69 are similar to those of E2, with the same allele group restrictions, B*35:01 and A*01:01 (B07 and A01 supertypes, respectively).

**E14 (SVFNVVNSSI):** This sequence was initially identified as A*02:01-restricted as the parent 15mer, C62, was positive with v01 (Table 4). Since there were not sufficient PBMC from v01, it was tested with v58 who shared the same HLA allele, A*02:01 (A02 supertype). E14 was also positive with v37 and NetMHC predicted binding to A*68:02 that like A*02:01 is part of the A02 supertype. E14 was also positive with v40 and NetMHC predicted a 9mer sequence **VFNVVNSSI** contained within E14 that is restricted by A*23:01 (A24 supertype). Therefore we conclude that E14 is restricted by A*02:01 and A*68:02 (both A02 supertype) and contains a sequence that is predicted to be restricted by A*23:01 (A24 supertype).

**E18(LIMVLSFLF):** This was positive with v156 and NetMHC predicted this sequence binds to A*29:02 (A01A24 supertype) that is expressed by v156. E18 was also positive with v37 and NetMHC predicted this sequence also binds to B*15:03 (B27 supertype). Finally, E18 was positive with v40, and NetMHC predicted binding to A*23:01 (A24 supertype). Therefore, E18 ELISpot activity was restricted by three allele groups, A*29:02, B*15:03 and A*23:01 that are members of three HLA supertypes, A01A24, B27 and A24, respectively. E17, which was not synthesized since several other A*02:01-restricted putative epitopes were tested (E1, E14, E20 and E22), has not yet been confirmed as A*02:01-restricted in ELISpot assays.

**E19(AILSVSSFLF):** This sequence was predicted as an A*23:01 (A24 supertype)-restricted epitope using v40. Since there were not sufficient PBMC from v40, E19 was tested and was positive with v63 who expresses A*24:03 that is also a member of the A24 supertype. NetMHC predicted that this 10mer was A*24:03-restricted (A24 supertype) with a low binding affinity (IC_50_ 1088 nM). Therefore, it was concluded that E18 may be restricted by A*23:01 and A*24:03, both of which are members of the A24 supertype.

**E20(SVSSFLFVEA):** This sequence was predicted to be A*68:02-restricted (A02 supertype) using v52. Since PBMC were not available from this volunteer, E20 was tested and was active with v125 who does not express A*68:02. However, NetMHC predicted that a 8mer contained within E20 **(SVSSFLFV)** is restricted by A*02:01 that is expressed by v125 and is also A02 supertype. Therefore E20 may be A*68:02-restricted, but ELISpot assay and NetMHC suggested that it contains a 8mer that is A*02:01-restricted (A02 supertype).

**E21(SFLFVEALF):** This sequence was positive with v37; NetMHC predicted binding to A*23:01 (A24 supertype) that is expressed by v37. E21 was also positive with v40 who also expressed the A*23:01 allele group. In addition, E21 was positive with v156 and NetMHC predicted binding to A*29:02 that is expressed by v156. E21 was also moderately positive with v68; NetMHC predicted binding to A*24:02 expressed by v68 that like A*23:01 is a member of the A24 supertype. Therefore, E21 appears to be restricted by A*23:01 and A*24:02 (both A24) and A*29:02 (A01A24).

**E22(IMVLSFLFL):** This sequence gave a modest response against v125 and NetMHC predicted that E22 is A*02:01-restricted as A*02:01 is expressed by v125. In addition, E22 partially overlaps a 10mer **LIMVLSFLFL** that is also predicted to be A*02:01-restricted consistent with v125 expressing the A02 supertype.

### Confirmation of class 1-restriction of epitopes using ELISpot depletion and ICS assays

PBMC from HLA-matched volunteers were available to conduct confirmatory assays for six of the nine predicted minimal epitopes tested: E1, E2, E14, E18, E20 and E21. After CD8+ T cell depletion, ELISpot activities against E1, E2, E18, E21 were reduced by 73%-96% (Table 6) whereas depletion of CD4+ T cells did not affect activity (reduction <18%). For E14, depletion of CD8+ and CD4+ T cells reduced activity approximately equally (56% and 50%, respectively). However, CD8+ T cell depletion did not affect activity of E20 whereas CD4+ T cell depletion reduced activity by 78%, suggesting that the response was not CD8+ T cell-dependent. When these epitopes were tested by ICS, results were consistent with the ELISpot depletion studies. CD8+ T cell interferon-gamma responses were recalled by E1, E2, E14, E18 and E21 at frequencies ranged from 0.12% (E14) to 0.54% (E18) of gated CD8+ T cells, similar to the parent pools, except for E20, where the CD8+ T cell frequency was only 0.06%. CD4+ T cell responses were negligible (≤0.04%). Therefore, five epitopes (E1, E2, E14, E18 and E21) of these six predicted epitopes were confirmed as minimal CD8+ T cell-dependent epitopes, while E20 was not.

**Table 6:** **ELISpot IFN**-**γ activity of CSP predicted epitopes after depletion of CD4**+ **and CD8**+ **T cells compared with ICS CD8**+ **and CD4**+ **T cell IFN**-**γ activity**

**Vol.**	**Pool**	**Epitope no.**	**Sequence**	**HLA allele group**	**Control depl. sfc/m**	CD8+ depl. sfc/m (%)^*^	CD4+ depl. sfc/m (%)^*^	CD8+ %^**^	CD4+ %^**^
V40^1^	Cp1				97	5 (-95%)	82 (-15%)	0.44	0.02
		**E21**	SFLFVEALF	A*23:01	254	15 (-94%)	226 (-11%)	0.37	0.04
		**E14**	SVFNVVNSSI	A*23:01	86	38 (-56%)	43 (-50%)	0.12	0.01
		**E18**	LIMVLSFLF	A*23:01	236	23 (-90%)	210 (-11%)	0.54	0.02
V69^2^	Cp1				445	6 (-98%)	397 (-11%)	0.53	0.01
		**E2**	FVEALFQEY	B*35:01	380	17 (-96%)	356 (-6%)	0.48	0.00
V125^3^		**E20**	SVSSFLFVEA	A*02:01	79	72 (-9%)	17 (-78%)	0.06	0.02
V156^3^	Cp1				133	0 (-100%)	135 (+2%)	0.33	0.01
		**E21**	SFLFVEALF	A*29:02	213	29 (-86%)	273 (+28%)	0.25	0.01
		**E1**	FLFVEALFQE	A*29:02	245	91 (-73%)	215 (-12%)	0.34	0.03
	Cp9	**E18**	LIMVLSFLF	A*29:02	307	85 (-82%)	264 (-18%)	0.26	0.01

^1^PBMC from one month after the first Ad-C immunization; ^2^PBMC from 19 days after the second Ad-C immunization; ^3^PBMC from 28 days after challenge following Ad-CA immunization.

*The percent change in ELISpot activity is shown after depletion of CD4+ or CD8+ T cells.

**The percent of CD8+ or CD4+ gated T cells expressing IFN-γ.

depl. = depletion.

sfc/m = spot forming cells/million.

v125 did not have sufficient PBMC to test Cp1.

### Summary of predicted and confirmed minimal CSP epitopes identified in this study

The confirmed minimal CSP epitopes are summarized in Table 7. Seventeen 9-10mer epitopes (E1–E17) were initially identified using NetMHC predictions of binding to the HLA A and B alleles expressed by a panel of Ad-CA and Ad-C-immunized volunteers (Tables 3 and 4). Five of these epitopes were synthesized (E1, E2, E3, E14 and E18) and tested in ELISpot assays. Four of these five epitopes were tested in ELISpot depletion and ICS assays (E1, E2, E14 and E18). Derived from strategy 2, four additional epitopes were synthesized (E19, E20, E21 and E22) and likewise demonstrated activity in ELISpot assays, and two of these were tested in ELISpot depletion and ICS assays (E20, E21). Overall, the restricted availability of PBMC from immunized volunteers allowed only five of these epitopes (E1, E2, E14, E18, and E21) to be confirmed as recalling CD8+ T cell responses. One additional epitope E20 was tested but could not be confirmed. All 9 of the putative epitopes that were synthesized and studied in ELISpot assays are conserved.

**Table 7:** Summary of predicted and confirmed minimal CSP identified in this study epitopes

**15mer peptide**	**Epitope number**	**Sequence**	**AA no.**	**15mer ELISpot activity**	**Epitope ELISpot activity**	**Depl. /ICS**	**HLA allele group**	**HLA supertype**
Cp1-C3	E1	FLFVEALFQE	13-22	+	+	+	A*02:01	A02
		FLFVEALFQE	13-21				A*29:02	A01A24
		FLFVEALFQE	13-20				A*29:02	A01A24
	E2	FVEALFQEY	15-23	+	+	ND	A*01:01	A01
		FVEALFQEY	15-23	+	+	+	B*35:01	B07
Cp1-C4	E3	LFVEALFQEY	14-23	+	+	ND	A*30:02	A01
Cp9-C62	E14	SVFNVVNSSI	376-385	+	+	ND	A*02:01	A02
					+		A*68:02	A02
		SVFNVVNSSI	377-385		+	+	A*23:01	A24
Cp9-C65	E18	LIMVLSFLF	387-395		+	+	A*23:01	A24
					+	+	A*29:02	A01A24
					+		B*15:03	B27
						ND		
Cp1-C1	E19	AILSVSSFLF	6-15		+	ND	A*24:03	A24
							A*23:01	A24
Cp1-C2	E20	SVSSFLFVEA	9-18		+	(+)	A*02:01	A02
Cp1-C3	E21	SFLFVEALF	12-20		+	+	A*23:01	A24
					+	+	A*29:02	A01A24
					+	ND	A*24:02	A24
Cp9-C65	E22	IMVLSFLFL	388-396		+	ND	A*02:01	A02

+ = Positive activity of the 15mer and predicted epitope in ELISpot assay, or recall of CD8+ T cells in ELISpot depletion (Depl.) assay or intracellular staining/flow cytometry (ICS) assay. (+) = Result not conclusive.

Underlined sequences indicate NetMHC-predicted putative class I-restricted epitopes within E1, E14 and E20.

The five epitopes with + in the Depl./ICS column are confirmed as CD8+ dependent.

### HLA allele promiscuity of identified epitopes using ELISpot assays

This study could not be extended to many subjects due to limited PBMC samples, but nevertheless supported previous findings that some class I epitopes are sufficiently degenerate to bind to more than one allele group or supertype [24]. As shown in Table 7, E2 was restricted by A*01:01 (A01 supertype) and B*35:01 (B07 supertype), and E18 was restricted by A*23:01 (A24 supertype), A*29:02 (A01A24 supertype) and B*1503 (B27 supertype). E14 was restricted by two allele groups of the A02 supertype (A*02:01 and A*68:02), and E21 was restricted by two allele groups of the A24 supertype (A*23:01 and A*24:02) and one allele group of the A01A24 supertype (A*29:02). This promiscuity of restriction is likely underestimated due to the limited availability of PBMC from the 17 volunteers used in this study. When NetMHC was used to predict all restrictions of epitopes within Cp1, Cp2, Cp6 and Cp9, many more potential restrictions were identified that could not be evaluated or verified in this study.

### Epitope localization

Among the 17 predicted putative epitopes, 11 were localized in the N-terminal region, and six were localized to the C-terminal region. Among the nine epitopes that were synthesized and tested in ELISpot assays, six were localized to the N-terminal region (E1, E2, E3, E19, E20, and E21) and three epitopes were localized to the C-terminal regions (E14, E18 and E22). Recently part of the strain 3D7 CSP C-terminal region (aa 310–375) has been crystallized revealing a unique αTSR domain related to other TSR domains that contains a hydrophobic pocket contiguous with the hydrophobic core [45]. Only one of the C-terminal epitopes described here completely lies within the crystallized sequence, E12 (Table 4), which is localized on an outer α1 helix that forms the edge of the hydrophobic pocket, and partially overlaps the Th2R epitope. However, E12 was not tested by itself although the two 15mers containing E12, C47 and C48, were tested in these assays. Predicted and not confirmed E15, E16 and E17 and confirmed E14, E18 and E22 localize to a short stretch (aa 376–396) that also contains the GPI anchor leading to the CSP C-terminus [45].

### Summary of functional epitopes within *Plasmodium falciparum* CSP

The summary of these newly identified epitopes, as well as those previously identified, is shown in Figure 1. Cp1, Cp2, Cp6 and Cp9 contain seven, eight, three and eight 15mer peptides, respectively, that overlap previously described A*02:01-restricted epitopes as shown in Figure 1. C1 overlaps D4 and C2 overlaps D3 but neither 15mer was positive by our criteria with volunteers expressing A*02:01 (see Additional file 1). C65 overlaps D2, and this 15mer was positive with v01 and v02 which do express A*02:01, and we identified an A*02:01-restricted epitope, E17, that overlaps D2, but E17 was not synthesized and tested in ELISpot assays. C47 and C48 overlap D5 that is A*02:01-restricted and both were positive in ELISpot assays with volunteers expressing A*02:01 (v61, v41, and v58, Table 4) and the NetMHC-predicted epitope, E12, is the same as D5. However, NetMHC also predicted E12/D5 to be B*08:01-restricted but E12 was not tested in the ELISpot assay with volunteers expressing A*02:01 or B*08:01.

## Discussion

Adenovirus-vectored vaccines (Ad-C and Ad-CA) are being developed to induce the CD8+ T cell responses thought to be required for protection against liver stage malaria [7]. The aim of this study was to better understand the cell-mediated immune responses targeting CSP elicited by these vaccines by mapping MHC class I restricted epitopes. The long-term goal was to aid the development of a broadly protective malaria vaccine for genetically diverse populations. To date, only a few class I-restricted epitopes have been described for CSP [24,27,46,47] and these earlier observations have now been extended by identifying additional class I-restricted epitopes.

As a first step, the computer algorithm NetMHC [48] was used to predict 11 putative minimal class 1-restricted epitopes within 15mer CSP peptides that were active in ELISpot assays conducted using PBMC from research volunteers immunized with the Ad-C- and Ad-CA-malaria vaccines. Although most HLA-restricted peptides have binding affinities of less than 50 nM, some may bind in the 50–500 nM range [43]. Therefore we focused on putative epitopes with predicted binding affinities of less than 500 nM. One of these epitopes, E12, has been previously described as D5 (Figure 1) specific for HLA A*02:02, although it was also predicted to bind to HLA B*08:01 in the studies reported here.

While peptide binding to class I MHC molecules is required for T cell recognition, many peptides that bind with high affinity are not recognized by T cells [33]. Therefore, it was necessary to demonstrate that these predicted epitopes were recognized by CD8+ T cells from Ad-C and Ad-CA-immunized volunteers. Five of the predicted epitopes were synthesized and tested with PBMCs from volunteers from the same clinical trials, and all were active as predicted with at least one HLA-matched volunteer. As a second approach, the direct prediction of putative class 1-restricted epitopes was pursued within parent pools that gave robust responses with selected volunteers without first screening individual 15mers in the ELISpot assay. Four of these epitopes were synthesized and tested, and again, all were active when tested with PBMC from HLA-matched volunteers. Thus NetMHC proved to be a valuable tool to predict epitopes within the parent pool to which the volunteers strongly responded with or without a preliminary screen of individual 15mers. Altogether, 18/20 matched epitope/volunteer pairings used to test the nine synthesized 9-10mers were positive on ELISpot assay. None of these nine putative epitopes has been previously described and all are therefore novel [49]. Having to rely on PBMC that were available meant that the evaluation could not be comprehensive, and indeed many more epitopes were predicted than could be tested.

It was possible to further test six of the nine synthesized epitopes by conducting additional ELISpot assays following CD4+ or CD8+ T cell depletion, and by conducting flow cytometry to phenotype the lymphocytes. Of the six epitopes tested, five demonstrated CD8+ T cell-dependent recall responses. These five also demonstrated a predominant CD8+ T cell response on flow cytometry. The fact that most class I-binding peptides are eight to 10 amino acids, while class II peptides range from 12 to 24 residues [24,50], supports the likelihood that the novel epitopes are class I-restricted.

Although testing of previously defined epitopes was not done, some 15mers tested contained previously identified epitopes. As one example, ELISpot activity was not demonstrated using two 15mers that overlap known A*02:01-restricted epitopes: C1 which overlaps D4, and C2 which overlaps D3. The reason for the lack of recall responses to these 15mers is unclear but probably it was not due to a false prediction by NetMHC, since comparative studies using NetMHC and other predictive algorithms found NetMHC to be the best performer across all HLA molecules, and particularly for predicting epitopes binding to A*02:01 molecules [51]. D4 and D3 were originally identified using PBMC from individuals living in a malaria-endemic area and therefore are recognized by naturally acquired immune CD8+ T cells, whereas in this study the Ad-C or Ad-CA vaccines may not induce the same responses as natural transmission. The lack of induced response might also have related to the fact that D4 and D3 lie within the signal sequence of CSP (aa 1–18, Figure 1) that may be cleaved during adenovirus expression of CSP in human cells and not efficiently processed and presented. A second example is provided by the C65 15mer that contains D2, which was also identified in individuals from malaria-endemic areas. In this case, unlike C1 and C2, C65 was positive in the ELISpot assay. This could reflect recognition of D2, or could also reflect other HLA-matched epitopes that were present in C65. Since D2, D3 and D4 were not tested as minimal epitopes, it was not possible to confirm whether a response mimicking naturally-acquired immunity, which responds to these epitopes, was induced by Ad-C or Ad-CA immunization. Future studies are planned to determine whether the novel epitopes identified in this study are also recognized by naturally-exposed individuals.

Of interest is that the RTS,S vaccine contains six previously described class I-restricted epitopes (D7, D1, D5, D6, D9 and D2) as well as three novel class I-restricted epitopes described here (E14, E18 and E22), yet, except for one observation [2], CD8+ T cell responses have not been described in RTS,S clinical trials [1]. The reasons for the lack of RTS,S-induced CD8+ T cell responses remain unclear but may be related to antigen presentation and processing of a protein-based vaccine as opposed to gene-based vaccines or may reflect the type of stimulants, such as the long synthetic peptides or recombinant proteins used to recall T-cell responses in some of the RTS,S immunological studies.

Earlier analyses of *P*. *falciparum* epitopes including CSP have suggested a high degree of degeneracy such that minimal 8-10mer peptides bind to more than one HLA allele within different supertypes [24]. This is consistent with findings that many different HLA alleles overlap in their peptide-binding properties [24,31-33,52-54]. Further examples were found in our study when NetMHC predictions of the HLA-restrictions of peptides spanning the full length of CSP were analysed (data not shown) suggesting that this HLA supertype promiscuity may be extensive. Promiscuous class I-restricted epitopes that recognize different HLA supertypes have been reported for viral diseases suggesting that many epitopes can be presented on different HLA alleles [38,39], with certain allele pairs frequently sharing epitopes [39]. In this study, the small number of epitopes formally tested precluded a comprehensive analysis. Nevertheless, the finding of degeneracy in the peptides that were studied provides encouraging evidence that a CSP adenovirus-vectored vaccine may be immunogenic in genetically diverse populations.

Broad applicability of the vaccine is supported by the lack of sequence variation observed in malaria-endemic areas for most of these epitopes, including all 9 that were synthesized [16,55,56]. Amino acid polymorphism may be associated with surface accessibility or immune pressure [56,57]. A large study involving isolates from Kenya, India, Cameroon and Venezuela identified only five polymorphic residues in the N-terminus of CSP [16,55]; one of these, a threonine, occurs in the E6 epitope described here. However, an analysis of the sequence of 3D7 CSP compared with nine other strains indicated that aa 1–63 containing E1-E11 as well as aa 371–396 containing E13-E22 are conserved, with only aa 324, 325 and 327, located within E12, showing variability. More frequent polymorphisms are found within the Th2R and Th3R epitopes, which do not overlap the epitopes described in this study [16,55,56]. More investigations are needed to determine the extent of polymorphism in class I-restricted epitopes, for example by comparison of sequenced genomes of strain 3D7 with endemic isolates using new technologies [58] that focus on CSP T cell epitopes [59].

Recent studies have suggested that the N-terminal region of CSP folds over and protects the C-terminal region, exposing the N-terminal and repeat regions [4]. CSP peptide pools containing peptides spanning the N-terminus recalled strong CD8+ T cell responses in Ad-C and Ad-CA-immunized volunteers. However, CSP peptide pools containing peptides spanning the C-terminal region recalled CD8+ T cell responses of similar magnitude, indicating that immune recognition is not related to localization within the protein sequence as found for other pathogens such as hepatitis C, HIV and influenza viruses [60]. Part of the N-terminal region containing E1, E2, E3, E19, E20 and E21 is proteolytically cleaved during sporozoite invasion, while truncated CSP containing E14, E18 and E22 is carried into the hepatocyte [4,45,61], suggesting that N- and C-terminal epitopes may be processed and presented to the immune system by different mechanisms. The epitopes identified here are contained within four immunodominant CSP peptide pools, and it is possible that immunodominance is influenced by differences in antigen processing [62].

## Conclusions

This study identified nine putative, conserved minimal epitopes of which five were confirmed as recalling CD8+ T cell responses. These are restricted by four HLA-A and two HLA-B supertypes that together are expressed by 99.5% of Caucasians and 98.1% African Americans [31]. Several of the CSP class I epitopes were found to be degenerate, recognized by multiple HLA alleles, consistent with prior reports [24]. Therefore it is likely that these adenovectored CSP vaccines will elicit CD8+ T cell responses in most Caucasian and African populations.

## Competing interests

DLD is an inventor listed on US Patent No., U.S. Patent No. 2009–0148477 A1, and international patent application PCT/ US06/33982, titled "Adenoviral Vector-based Malaria Vaccines"; TLR and DLD are inventors listed on US Patent Application 12/522,335, and international patent application PCT/US08/50565 titled "Adenoviral Vector-based Malaria Vaccines".

## Authors’ contributions

MS designed research; MS, HG, JL, EA, GB, and MB performed ELISpot assays; FF, JH, and SM performed the ICS assays; YK, BP and AS used NetMHC to predict epitopes; TLR, EV, DLD, CD and LS provided intellectual input. CT was an investigator in the clinical trial; MS, YK, BP, DLD, EV, MRH and TLR wrote the paper. All authors read and approved the final manuscript.

## Supplementary Material

Additional file 1 ELISpot IFN-γ activity of CSP peptide pools and individual 15-mer peptides within these pools with Ad-CA and Ad-C-immunized volunteers (Strategy 1). Click here for file

Additional file 2 ELISpot IFN-γ activity of synthesized predicted putative epitopes with Ad-CA and Ad-C-immunized volunteers (Strategies 1 and 2). Click here for file
