# Supplementary material for: Identification of minimal human MHC-restricted CD8+ T-cell epitopes within the Plasmodium falciparum circumsporozoite protein (CSP)
Source: Malar J. 2013 Jun 5;12:185. doi: 10.1186/1475-2875-12-185 (PMC3683343; doi:10.1186/1475-2875-12-185)
Supplement: Additional file 1 — ELISpot IFN-γ activity of CSP peptide pools and individual 15-mer peptides within these pools with Ad-CA and Ad-C-immunized volunteers (Strategy 1). [file 1475-2875-12-185-S1.docx]

**Additional File 1: ELISpot IFN-γ activity of CSP peptide pools and individual 15-mer peptides within these pools with Ad-CA and Ad-C-immunized volunteers (Strategy 1)**

|  | | | **Vol.** | **V41** | | **V49** | | | **v58** | | **V61** | | **V69** | | **V01** | | **V02** | | **V05** | | **V08** | | **V12** | |
| --- | --- | --- | --- | --- | --- | --- | --- | --- | --- | --- | --- | --- | --- | --- | --- | --- | --- | --- | --- | --- | --- | --- | --- | --- |
|  |  |  | **A Allele** | **A*02:01**  A*31:01 | | A*33:01  A*74:01 | | | **A*02:01**  A*24:02 | | **A*02:01**  **A*02:01** | | A*30:02  A*34:02 | | **A*02:01**  A*26:01 | | A*01:01  **A*02:01** | | **A*01:01**  A*68:02 | | A*68:01  **A*68:02** | | **A*30:02**  A*68:01 | |
|  |  |  | **A ST** | **A02**/A03 | | A03/A03 | | | **A02**/A24 | | **A02/A02** | | A01/A03 | | **A02**/A01 | | A01/**A02** | | **A01**/A02 | | A03/**A02** | | **A01**/A03 | |
|  |  |  | **B Allele** | B*0702  **B*3501** | | **B*15:03**  **B*15:03** | | | B*08:01  B*38:02 | | B*38:01  **B*44:02** | | B*14:02  **B*35:01** | | B*18:01  **B*44:02** | | B*08:01  B*44:02 | | **B*08:01**  B*14:02 | | B*14:02  B*48:01 | | B*18:01  B*58:02 | |
|  |  |  | **B ST** | B07/**B07** | | **B27/B27** | | | B08/B27 | | B27/**B44** | | B27/**B07** | | B44/**B44** | | B08/B44 | | **B08**/B27 | | B27/B27 | | B44/B58 | |
|  |  |  |  | **IC**  **_50_** | **Sfc/m** | **IC**  **_50_** | **Sfc/m** | | **IC**  **_50_** | **Sfc/m** | **IC**  **_50_** | **Sfc/m** | **IC**  **_50_** | **Sfc/m** | **IC**  **_50_** | **Sfc/m** | **IC**  **_50_** | **Sfc/m** | **IC**  **_50_** | **Sfc/m** | **IC**  **_50_** | **Sfc/m** | **IC**  **_50_** | **Sfc/m** |
| **Cp1** | **Pool of C1-C7** |  |  |  | 12 |  |  | |  | **116** |  | 23 |  | **411** |  | **77** |  |  |  | **142** |  |  |  | **64** |
|  | **Sequence** | **Allele** | **ST** |  |  |  |  | |  |  |  |  |  |  |  |  |  |  |  |  |  |  |  |  |
| **C1** | MMRKLAILSVSSFLF | A*02:01 | A02 | 28 | 10 |  |  | | 28 | 15 | 28 | 17 |  |  | 28 | 0 | 28 | 30 |  |  |  |  |  |  |
|  | MMRKLAILSVSSFLF | B*08:01 | B08 |  |  |  |  | |  |  |  |  |  |  |  |  |  |  | 97 | 27 |  |  |  |  |
|  | MMRKLAILSVSSFLF | B*35:01 | B07 |  |  |  |  | |  |  |  |  | 451 | 0 |  |  |  |  |  |  |  |  |  |  |
| **C2** | LAILSVSSFLFVEAL | A*02:01 | A02 | 23 | 7 |  |  | | 23 | 12 | 23 | 0 |  |  | 23 | 0 | 23 | 24 |  |  |  |  |  |  |
|  | LAILSVSSFLFVEAL | B*35:01 | B07 |  |  |  |  | |  |  |  |  | 451 | 8 |  |  |  |  |  |  |  |  |  |  |
|  | LAILSVSSFLFVEAL | A*68:02 | A02 |  |  |  |  | |  |  |  |  |  |  |  |  |  |  | 104 | 21 | 104 | 15 |  |  |
| **C3** | SVSSFLFVEALFQEY | **B*35:01** | **B07** | 68 | 17 |  |  | |  |  |  |  | 68 | **385** |  |  |  |  |  |  |  |  |  |  |
|  | SVSSFLFVEALFQEY | **A*01:01** | **A01** |  |  |  |  | |  |  |  |  |  |  |  |  | 50 | 0 | 50 | **65** |  |  |  |  |
|  | SVSSFLFVEALFQEY | **A*02:01** | **A02** |  |  |  |  | | 258 | **29** | 258 | 0 |  |  | 258 | 0 |  |  |  |  |  |  |  |  |
|  | SVSSFLFVEALFQEY | A*30:02 | A01 |  |  |  |  | |  |  |  |  |  |  |  |  |  |  |  |  |  |  | 226 | 29 |
|  | SVSSFLFVEALFQEY | A*68:02 | A02 |  |  |  |  | |  |  |  |  |  |  |  |  |  |  |  |  | 104 | 12 |  |  |
| **C4** | FLFVEALFQEYQCYG | **B*35:01** | **B07** | 68 | 25 |  |  | |  |  |  |  | 68 | **368** |  |  |  |  |  |  |  |  |  |  |
|  | FLFVEALFQEYQCYG | **A*01:01** | **A01** |  |  |  |  | |  |  |  |  |  |  |  |  | 50 | 0 | 50 | **44** |  |  |  |  |
|  | FLFVEALFQEYQCYG | **A*02:01** | **A02** |  |  |  |  | | 258 | 43 | 258 | 13 |  |  | 258 | **48** |  |  |  |  |  |  |  |  |
|  | FLFVEALFQEYQCYG | **A*30:02** | **A01** |  |  |  |  | |  |  |  |  |  |  |  |  |  |  |  |  |  |  | 226 | **87** |
| **C5** | EALFQEYQCYGSSSN | A*30:02 | A01 |  |  |  |  | |  |  |  |  | 296 | 21 |  |  |  |  |  |  |  |  | 296 | 3 |
|  | EALFQEYQCYGSSSN | **A*02:01** | **A02** |  |  |  |  | | 2174 | **105** |  |  |  |  |  |  |  |  |  |  |  |  |  |  |
| **C6** | QEYQCYGSSSNTRVL | A*31:01 | A03 | 170 | 3 |  |  | |  |  |  |  |  |  |  |  |  |  |  |  |  |  |  |  |
|  | QEYQCYGSSSNTRVL | **A*02:01** | **A02** |  |  |  |  | | >10^5^ | **83** |  |  |  |  |  |  |  |  |  |  |  |  |  |  |
|  | | | | | | | | | | | | | | | | | | | | | | | | |
| **Cp2** | **Pool of C8-C15** |  |  |  | **83** |  | | **64** |  |  |  |  |  |  |  | **119** |  | 38 |  | 18 |  |  |  | **331** |
| **C8** | SSNTRVLNELNYDNA | A*01:01 | A01 |  |  |  | |  |  |  |  |  |  |  |  |  | 297 | 0 | 297 | 12 |  |  |  |  |
|  | SSNTRVLNELNYDNA | **B*15:03** | **B27** |  |  | 4075 | | **41** |  |  |  |  |  |  |  |  |  |  |  |  |  |  |  |  |
| **C10** | ELNYDNAGTNLYNEL | **B*35:01** | **B07** | 321 | **35** |  | |  |  |  |  |  |  |  |  |  |  |  |  |  |  |  |  |  |
|  | ELNYDNAGTNLYNEL | A*01:01 | A01 |  |  |  | |  |  |  |  |  |  |  |  |  | 299 | 13 | 299 | 24 |  |  |  |  |
| **C12** | TNLYNELEMNYYGQ | **B*15:03** | **B27** |  |  | 1087 | | **28** |  |  |  |  |  |  |  |  |  |  |  |  |  |  |  |  |
|  | TNLYNELEMNYYGKQ | A*01:01 | A01 |  |  |  | |  |  |  |  |  |  |  |  |  | 114 | 8 | 114 | 0 |  |  |  |  |
|  | TNLYNELEMNYYGQ | **A*30:02** | **A01** |  |  |  | |  |  |  |  |  |  |  |  |  |  |  |  |  |  |  | 25 | **411** |
|  | TNLYNELEMNYYGQ | **B*44:02** | **B44** |  |  |  | |  |  |  |  |  |  |  | 468 | **119** |  |  |  |  |  |  |  |  |
| **C13** | NELEMNYYGKQENWY | **B*44:02** | **B44** |  |  |  | |  |  |  |  |  |  |  | 468 | **116** |  |  |  |  |  |  |  |  |
|  | NELEMNYYGKQENWY | **A*30:02** | **A01** |  |  |  | |  |  |  |  |  |  |  |  |  |  |  |  |  |  |  | 132 | **334** |
| **C14** | MNYYGKQENWYSLKK | A*31:01 | A03 | 93 | 3 |  | |  |  |  |  |  |  |  |  |  |  |  |  |  |  |  |  |  |
| **C15** | GKQENWYSLKKNSRS | A*31:01 | A03 | 53 | 20 |  | |  |  |  |  |  |  |  |  |  |  |  |  |  |  |  |  |  |
|  | | | | | | | | | | | | | | | | | | | | | | | | |
| **Cp6** | **Pool of C46-C48** |  |  |  | **95** |  | |  |  | 24 |  | **53** |  |  |  | 31 |  | 47 |  | **130** |  |  |  |  |
| **C46** | EEPSDKHIKEYLNKI | B*08:01 | B08 |  |  |  | |  | 170 | 0 |  |  |  |  |  |  | 170 | 2 | 170 | 3 |  |  |  |  |
|  | EEPSDKHIKEYLNKI | A*26:01 | A01 |  |  |  | |  |  |  |  |  |  |  | 98 | 0 |  |  |  |  |  |  |  |  |
| **C47** | DKHIKEYLNKIQNSL | **A*02:01** | **A02** | 27 | 16 |  | |  | 27 | 30 | 27 | **48** |  |  | 27 | 2 | 27 | 19 |  |  |  |  |  |  |
|  | DKHIKEYLNKIQNSL | B*08:01 | B08 |  |  |  | |  |  |  |  |  |  |  |  |  |  |  | 83 | 38 |  |  |  |  |
| **C48** | KEYLNKIQNSLSTEW | **A*02:01** | **A02** | 27 | **103** |  | |  | 27 | **45** | 27 | 22 |  |  | 27 | 8 | 27 | 2 |  |  |  |  |  |  |
|  | KEYLNKIQNSLSTEW | **B*08:01** | **B08** |  |  |  | |  |  |  |  |  |  |  |  |  |  |  | 83 | **106** |  |  |  |  |
|  | | | | | | | | | | | | | | | | | | | | | | | | |
| **Cp9** | **Pool of C58-C65** |  |  |  | 21 |  | |  |  |  |  | 128 |  |  |  | **39** |  | 16 |  | 80 |  | **142** |  |  |
| **C58** | ELDYANDIEKKICKM | A*68:01 | A03 |  |  |  | |  |  |  |  |  |  |  |  |  |  |  |  |  | 370 | 0 |  |  |
| **C60** | EKKICKMEKCSSVFN | B*08:01 | B08 |  |  |  | |  |  |  |  |  |  |  |  |  | 60 | 5 | 60 | 12 |  |  |  |  |
|  | EKKICKMEKCSSVFN | **B*44:02** | **B44** |  |  |  | |  |  |  | 2353 | **39** |  |  |  |  |  |  |  |  |  |  |  |  |
| **C61** | CKMEKCSSVFNVVNS | B*08:01 | B08 |  |  |  | |  |  |  |  |  |  |  |  |  | 60 | 2 | 60 | 0 |  |  |  |  |
| **C62** | KCSSVFNVVNSSIGL | **A*02:01** | **A02** | 470 | 6 |  | |  |  |  | 470 | 8 |  |  | 470 | **116** | 470 | 6 |  |  |  |  |  |  |
|  | KCSSVFNVVNSSIGL | A*68:02 | A02 |  |  |  | |  |  |  |  |  |  |  |  |  |  |  | 18 | 6 | 18 | 6 |  |  |
| **C63** | VFNVVNSSIGLIMVL | **A*68:02** | **A02** |  |  |  | |  |  |  |  |  |  |  |  |  |  |  | 70 | 6 | 70 | **109** |  |  |
|  | VFNVVNSSIGLIMVL | B*15:01 | B62 |  |  |  | |  |  |  |  |  |  |  |  |  |  |  | 294 | 9 |  |  |  |  |
|  | VFNVVNSSIGLIMVL | **A*02:01** | **A02** |  |  |  | |  |  |  | 2083 | 80 |  |  |  |  |  |  |  |  |  |  |  |  |
| **C64** | VNSSIGLIMVLSFLF | **A*68:02** | **A02** |  |  |  | |  |  |  |  |  |  |  |  |  |  |  |  |  | 294 | **91** |  |  |
|  | VNSSIGLIMVLSFLF | A*02:01 | A02 |  |  |  | |  |  |  |  |  |  |  | 93 | 4 | 93 | 5 |  |  |  |  |  |  |
|  | VNSSIGLIMVLSFLF | A*02:01 | A02 | 93 | 2 |  | |  |  |  | 93 | 51 |  |  |  |  |  |  |  |  |  |  |  |  |
| **C65** | SSIGLIMVLSFLFLN | **A*02:01** | **A02** | 53 | 1 |  | |  |  |  | 53 | 58 |  |  | 53 | **46** | 53 | **44** |  |  |  |  |  |  |
|  | SSIGLIMVLSFLFLN | **A*68:02** | **A02** |  |  |  | |  |  |  |  |  |  |  |  |  |  |  |  |  | 816 | **62** |  |  |

All 15mers contained within Cp1, Cp2, Cp6 and Cp9 were tested in ELISpot assay with five volunteers immunized with Ad-CA (without CHMI) and five volunteers immunized with Ad-C. Positive assays are shown in bold. Matched HLA allele groups and HLA supertypes (ST) of the epitopes and volunteers are also shown in bold. Fifteen of the 15mers were positive with one or more HLA-matched volunteers.
