# Supplementary material for: Identification of minimal human MHC-restricted CD8+ T-cell epitopes within the Plasmodium falciparum circumsporozoite protein (CSP)
Source: Malar J. 2013 Jun 5;12:185. doi: 10.1186/1475-2875-12-185 (PMC3683343; doi:10.1186/1475-2875-12-185)
Supplement: Additional file 2 — ELISpot IFN-γ activity of synthesized predicted putative epitopes with Ad-CA and Ad-C-immunized volunteers (Strategies 1 and 2). [file 1475-2875-12-185-S2.docx]

**Additional File 2. ELISpot IFN-γ activity of synthesized predicted putative epitopes with Ad-CA and Ad-C-immunized volunteers (Strategies 1 and 2)**

|  | | | | **Vol.** | **V37** | | **V40** | | | **V58** | | **V63** | | **V68** | | **V69** | | **V125** | | **V127** | | **V156** | |
| --- | --- | --- | --- | --- | --- | --- | --- | --- | --- | --- | --- | --- | --- | --- | --- | --- | --- | --- | --- | --- | --- | --- | --- |
|  |  |  |  | **A Allele** | **A*23:01**  **A*68:02** | | **A*23:01**  A*29:02 | | | **A*02:01**  A*24:02 | | A*11:01  **A*24:03** | | **A*24:02**  A*30:01 | | A*30:02  A*34:02 | | **A*02:01**  A*11:01 | | **A*01:01**  A*24:02 | | **A*03:01**  **A*29:02** | |
|  |  |  |  | **A ST** | **A24/A02** | | **A24**/A01 | | | **A02**/A24 | | A03/**A24** | | **A24**/A01A03 | | A01/A03 | | **A02**/A03 | | **A01**/**A24** | | **A03**/**A01A24** | |
|  |  |  |  | **B Allele** | **B*15:03**  B*53:01 | | B*52:01  B*53:01 | | | B*08:01  B*38:02 | | B*40:01  B*51:04 | | B*13:02  B*1402 | | B*14:02  **B*35:01** | | B*35:01  B*52:01 | | B*08:01  B*44:05 | | B*15:03  B*58:02 | |
|  |  |  |  | **B ST** | **B27/**B07 | | B62/B07 | | | B08/B27 | | B44/B07 | | B62/B27 | | B27/**B07** | | B07/B62 | | B08/B44 | | B27/B58 | |
|  |  |  |  |  | IC_50_ | Sfc/m | IC_50_ | Sfc/m | | IC_50_ | Sfc/m | IC_50_ | Sfc/m | IC_50_ | Sfc/m | IC_50_ | Sfc/m | IC_50_ | Sfc/m | IC_50_ | Sfc/m | IC_50_ | Sfc/m |
| **Pool** | **Epitope** | **Sequence** | **Allele** | **ST** |  |  |  |  | |  |  |  |  |  |  |  |  |  |  |  |  |  |  |
| **Cp1** | **E1** | FLFVEALFQE | **A*02:01** | **A02** |  |  |  |  | | 258 | **49** |  |  |  |  |  |  |  |  |  |  |  |  |
|  | **E1** | FLFVEALFQE | **A*29:02** | **A01A24** |  |  |  |  | |  |  |  |  |  |  |  |  |  |  |  |  | 422 | **105** |
|  | **E1** | FLFVEALFQE | **A*29:02** | **A01A24** |  |  |  |  | |  |  |  |  |  |  |  |  |  |  |  |  | 109 | **105** |
|  | **E2** | FVEALFQEY | **B*35:01** | **B07** |  |  |  |  | |  |  |  |  |  |  | 68 | **268** |  |  |  |  |  |  |
|  | **E2** | FVEALFQEY | **A*01:01** | **A01** |  |  |  |  | |  |  |  |  |  |  |  |  |  |  | 63 | **89** |  |  |
|  | **E3(E2)** | LFVEALFQEY | **A*01:01** | **A01** |  |  |  |  | |  |  |  |  |  |  |  |  | NP | 14 | 63 | **99** | NP | 28 |
|  | **E3(E2)** | LFVEALFQEY | **B*35:01** | **B07** |  |  |  |  | |  |  |  |  |  |  | 68 | **259** |  |  |  |  |  |  |
|  | **E19** | AILSVSSFLF | A*23:01 | A24 |  |  | 192 | 14 | | NP | 46 |  |  |  |  |  |  |  |  |  |  |  |  |
|  | **E19** | AILSVSSFLF | **A*24:03** | **A24** |  |  |  |  | |  |  | 1088 | **41** |  |  |  |  |  |  |  |  |  |  |
|  | **E20** | SVSSFLFVEA | **A*02:01** | **A02** |  |  |  |  | |  |  |  |  |  |  |  |  | 25 | **33** |  |  |  |  |
|  | **E21** | SFLFVEALF | **A*29:02** | **A01A24** |  |  |  |  | |  |  |  |  |  |  |  |  |  |  | NP | 6 | 245 | **240** |
|  | **E21** | SFLFVEALF | **A*23:01** | **A24** | 104 | **53, 53** | 104 | **313, 197** | | NP | 21 |  |  |  |  | NP | 0 |  |  |  |  |  |  |
|  | **E21** | SFLFVEALF | **A*24:02** | **A24** |  |  |  |  | |  |  |  |  | 104 | **34** |  |  |  |  |  |  |  |  |
|  | |  | | | | | | | | | | | | | | | | | | | | | |
| **Cp9** | **E14** | SVFNVVNSSI | **A*68:02** | **A02** | 18 | **28** |  |  |  | |  |  |  |  |  | NP | 0 |  |  |  |  |  |  |
|  | **E14** | SVFNVVNSSI | **A*02:01** | **A02** |  |  |  |  | 470 | | **80** |  |  |  |  |  |  |  |  |  |  |  |  |
|  | **E14** | SVFNVVNSSI | **A*23:01** | **A24** |  |  | 1801 | **104**  **72** |  | |  |  |  |  |  |  |  |  |  |  |  |  |  |
|  | **E18** | LIMVLSFLF | **A*29:02** | **A01A24** |  |  |  |  |  | |  |  |  |  |  |  |  |  |  |  |  | 190 | **156** |
|  | **E18** | LIMVLSFLF | **B*15:03** | **B27** | 111 | **55**  **38** |  |  | NP | | 47 | NP | 1 |  |  | 224 | 0 |  |  |  |  |  |  |
|  | **E18** | LIMVLSFLF | **A*23:01** | **A24** |  |  | 282 | **290**  **166** |  | |  |  |  |  |  |  |  |  |  |  |  |  |  |
|  | **E22** | IMVLSFLFL | **A*02:01** | **A02** |  |  |  |  |  | |  |  |  |  |  |  |  | 59 | **29** |  |  |  |  |
|  | **E22** | IMVLSFLFL | B*15:03 | B27 | 13 | 15 | NP | 18 | NP | | 2 | NP | 0 |  |  | NP | 0 |  |  |  |  |  |  |

Five epitopes identified by screening CSP 15mers (Strategy 1, Additional File 1, Tables 3, 4 and 5), and four epitopes identified in the same 15mers using NetMHC to identify minimal epitopes matched to additional volunteers (Strategy 2, Table 5), were tested in ELISpot assay using five volunteers immunized with Ad-C and three immunized with Ad-CA followed by challenge. Positive assays are shown in bold. Matched HLA allele groups and HLA supertypes (ST) of the epitopes and volunteers are shown in bold. E3 (E2) indicates that NetMHC identified E2 contained within E3.

U=supertype not assigned; NP = Not predicted
